# Polypharmacy in Older Patients with Multimorbidity: The Agreement Between Patient and General Practitioner-Reported Drugs Observed in a Pilot cRCT

**DOI:** 10.3390/ijerph21101389

**Published:** 2024-10-21

**Authors:** Lena Schäfer, Michael Paulitsch, Maria Hanf, Truc Sophia Dinh, Astrid-Alexandra Klein, Sophia Klasing, Hanna Seidling, Karen Voigt, Marjan van den Akker

**Affiliations:** 1 Institute of General Practice, Goethe University, Theodor-Stern-Kai 7, 60590 Frankfurt am Main, Germanym.vandenakker@allgemeinmedizin.uni-frankfurt.de (M.v.d.A.); 2 Department of General Practice, Faculty of Medicine and University Hospital Carl Gustav Carus, TUD Dresden University of Technology, Fetscherstraße 74, 01307 Dresden, Germany; 3 Cooperation Unit Clinical Pharmacy, Internal Medicine IX-Department of Clinical Pharmacology and Pharmacoepidemiology, Medical Faculty Heidelberg, Heidelberg University Hospital, Heidelberg University, Im Neuenheimer Feld 410, 69120 Heidelberg, Germanyhanna.seidling@med.uni-heidelberg.de (H.S.)

**Keywords:** polypharmacy, multimorbidity, primary care, older patients, pilot study, medication management

## Abstract

Polypharmacy (≥5 drugs) increases the risk of discrepancies between patient- and general practitioner (GP)-reported drugs, leading to adverse outcomes. This explorative analysis assesses the agreement between patient- and GP-reported drugs under the influence of a paper-based patient portfolio in a pilot cluster randomized controlled trial (cRCT). Complete data were available for 68 patients aged 65 or older (26 were female), with multimorbidity, polypharmacy, and at least one hospitalization in the past year. Agreement was assessed for drug name and strength level. Differences between the intervention and control group (IG/CG) and comparisons between two time points (six-month interval) stratified according to gender were analyzed using Wilcoxon and Mann–Whitney U tests (α = 5%). To evaluate the reasons for discrepancies, the agreement of active pharmaceutical ingredients (APIs) and anatomical therapeutic chemical (ATC) groups was analyzed. At baseline, the agreement was 72.1% for the IG and 73.9% for the CG. Inclusion of the reported drug strength reduced the agreement in both groups (IG 66.7%, CG 60.0%). Agreement for the IG decreased statistically significantly after six months (−5.4%). ATC groups B, C, and H had the highest agreement, while N, R, and Z had the lowest. Large discrepancies in the drugs reported, due to the APIs and the corresponding ATC group, were observed.

## 1. Introduction

The challenge of polypharmacy associated with aging is recognized by society as a whole. From the age of 70, there is a significant increase in the proportion of patients who are prescribed multiple drugs [1]. The use of five or more drugs at a time is commonly described as polypharmacy [2]. Taking numerous drugs can result in adverse effects, drug–drug interactions, and drug–disease interactions [3,4,5]. The free choice of doctors, including specialists, regulated by law in Germany, can additionally increase the number of medications prescribed and taken [6]. Due to the absence of an electronically available patient file or medication list and insufficient medication and health information, medication errors, a low quality of care, and risks for patient safety arise [7,8]. Patients often take drugs not listed in their patient files, especially when it comes to over-the-counter (OTC) drugs. The fact that these drugs can be easily purchased from online stores increases the risk of missing information about the drugs a patient is taking. Coordinating comprehensive health information, including diseases, medication lists, or clinic visit summaries, is challenging for general practitioners (GPs) and all other healthcare providers [9,10,11,12]. Also, the gap in communication between patients and general practitioners regarding medication adherence, adverse effects, and drug-related problems is a high risk for medication safety [12,13]. Poor communication between healthcare providers could worsen the problems related to polypharmacy, with drug–drug interactions, drug failure, and hepatic damage being the most common [9]. This issue has been evaluated in various studies. One study showed that in patients with polypharmacy, the patient and GP agreed in only 56% of cases on the number of drugs [14]. Another study, which also included OTC drugs, found that the GP, pharmacist, and patient were in complete agreement on prescribed drugs in only 60.4% of cases [15]. Additionally, it was found in a previous study that in 95.8% of cases, there was at least one discrepancy between the patient’s and GP’s report, with most discrepancies resulting from OTC drugs purchased by the patient without a prescription [9].

In a prospective, cluster randomized, multicenter pilot study on HYPERION-TransCare (‘Heading to ContinuitY of Prescribing in EldeRly with MultImOrbidity iN Transitional Care’), the feasibility of the study design and a new complex intervention were evaluated. The study aimed to reduce negative health outcomes by enhancing the continuity of information between patients and healthcare providers.

This paper exploratively analyzed agreement between patient- and GP-reported drugs, with a focus on the following research questions.

1. What is the agreement about the drugs reported by patients and those reported by GPs, in relation to the drug name and strength?
2. Does agreement improve after introducing a paper-based patient portfolio?
3. Are there differences in drug agreement between gender subgroups?
4. Are there differences in drug agreement between single active pharmaceutical ingredients (APIs) or anatomical therapeutic chemical (ATC) groups?

## 2. Materials and Methods

### 2.1. Data Collection

Data were collected in the HYPERION-TransCare study [16], a prospective, cluster randomized, multicenter pilot study with two parallel groups. The study was registered in the DRKS German Clinical Trials Register: DRKS000276 49 (date: 19 January 2022). The study was conducted in accordance with the Declaration of Helsinki and approved by the Institutional Ethics Committee of the Technical University of Dresden (SR-EK-550112021), the Ethics Committee of the Chamber of Physicians of Saxony (EK-BR-14/22-1), and the Department of Medicine at Goethe University Frankfurt (2021-472); the process of evaluation was approved by the Ethics Committee of the Medical Faculty of Heidelberg University (S-068/2022). The intervention period was six months, with data collection at baseline (t0) and after six months of follow-up (t1). The study period was from March to December 2022 and was conducted in GP practices in the region of Hesse and Saxony (Germany). The patient inclusion criteria were as follows: age 65 years or older, two or more chronic diseases, five or more chronic drugs, at least one hospitalization in the past 12 months, and sufficient knowledge of German. Patients who were unable to give written consent, resided in a nursing home, or had been diagnosed with severe mental disorders (ICD-10 F diagnoses) were excluded. The randomization was performed by a project-independent person at the Goethe University Frankfurt at the level of practices (clusters) to avoid contamination among patients. An online tool [17] generated a random sequence of the two study arms in blocks of 6 and 4, specific to each study region. Each practice was assigned with a consecutive pseudonym and added to the randomization list in the order that patients from that practice completed their baseline surveys (t0). As a result, each practice was randomly assigned to one of the study arms. The intervention group (IG) received a paper-based patient portfolio to enhance the continuity of drug data between patients and their GPs. The patient portfolio is described in more detail in the study protocol [16]. The patient portfolio was to be completed with the GP’s support and was structured as follows: 1. patient-individual data, 2. medication data, 3. disease data, 4. legal documents, 5. relevant notes, 6. hospital transition information, and 7. other information [16]. In addition to the portfolio, patients were given a flyer with general guidance on communicating information with healthcare providers, especially regarding drug information. Patients were instructed and encouraged to take their patient portfolio to every GP appointment or hospital stay and add new information after each visit. IG practices were given a ‘GP practice checklist’ that reflected information from the patient portfolio and the German S3 guideline on multimedication [18]. Patients in the CG only received the patient flyer to ensure adequate blinding. CG practices were also provided with the S3 guideline on multimedication [18].

Patient-reported data were collected using structured written questionnaires at t0 and t1. The patient questionnaire was structured as follows: 1. personal data, 2. health status (with questions about physical symptoms in the last seven days [19]), and 3. the use of health services using the FIMA questionnaire (Fragebogen zur Inanspruchnahme medizinischer und nicht-medizinischer Versorgungsleistungen im Alter [Questionnaire for Health-Related Resource Use in an Elderly Population]), which is a validated tool for collecting health-related resource use within an older population [20,21]. The patient was requested to report the drug name, German pharmaceutical registration number (PZN), package size, and dosage of drugs taken in the previous seven days (note on OTC drugs). Structured digital questionnaires were used for GP-reported data collection at t0 and t1 via the electronic data capture system Research Electronic Data Capture (REDCap) (hosted at the Technical University of Dresden [22,23]). The GP questionnaires included the following: 1. patient diseases as classed in 14 categories based on the Cumulative Illness Rating Scale (CIRS) (CIRS data were only queried at time t0) [24]; 2. according to the FIMA questionnaires, the number of visits to a GP practice and other healthcare providers in the last three months, drug data of the previous seven days, inpatient stays in the last 12 months, and health insurance status and living arrangement. Either the GP or a healthcare assistant (HCA) could complete the questionnaires using data stored within the practice.

### 2.2. Data Analysis

Sociodemographic data of GPs and patients were analyzed using the chi-square test with α = 5% and a 95% confidence interval (CI).

The absolute agreement was determined by comparing patient-reported and GP-reported drug names. The reported drug names were matched by comparing strings. The reported drug strength was also matched, and the absolute agreement based on the drug name, including drug strength, was calculated. To determine agreement between patient-reported and GP-reported drugs, an R-script was written using the package tidyverse [25]. This allowed for a very accurate comparison of drug names and reported strengths. In addition, the method is quick and efficient and provides reproducible results. Only data from patients where the patient and GP reported t0 and t1 drug data were used.

The relative agreement calculation compared the absolute agreement based on the reported number of drugs. Therefore, the level of agreement was calculated as the ratio of absolute agreement to the total number of agreements and disagreements per patient. Both outcome variables, the relative agreement of drug name and the relative agreement of drug name including drug strength, were analyzed at two time points: baseline (t0) and six-month follow-up (t1). The statistical analysis and graph creation were conducted using IBM SPSS Statistics SPSS Inc., Chicago, Ill., USA (Version: 28.0.1.1). The Mann–Whitney U test was used to evaluate group differences between the IG and CG and gender subgroups of the IG and CG, and the Wilcoxon test was performed to analyze differences between t0 and t1. Both tests used α = 5% and a 95% confidence interval (CI).

A sensitivity analysis was performed regarding drug name and drug strength at t0 and t1, excluding OTC drugs. The PZN, or the reported strength of the product, was used to distinguish between OTC and prescription-only drugs. If only the API was reported, the presence of a prescription-only drug product was checked. Therefore, OTC drugs that did not have a corresponding prescription-only drug product were excluded. For example, ibuprofen with a product strength of 400 mg does not require a prescription (in Germany) and was excluded from the sensitivity analysis, but ibuprofen with a product strength of 600 mg or more per tablet does require a prescription and was included.

Further, the agreement of single APIs at t0 and t1 was analyzed. For this, the corresponding API for the drug product was transcribed. The number of matches between patient-reported API and GP-reported API determined the agreement at the drug level. The ratio of absolute agreement to the total number of agreements and disagreements was calculated to average the agreement. By looking at all drugs that were reported by the patient and GP, only drugs with ten or more reports (GP level or patient level) were included to ensure an adequate database.

All APIs were allocated to ATC groups (1st level) A-Z [26]. The ATC classification system is an international system that determines APIs based on their anatomical, therapeutic, and chemical usage. The absolute number of additions, deletions, and agreements of all patients related to the ATC group was counted. Additions are drugs that the patient additionally reported, and deletions are anything reported on the GP’s list but not reported by the patient. The percentage of additions, deletions, and agreements was calculated, in each case, as the ratio of the absolute number of each to the sum of all reported drugs in the respective ATC group.

## 3. Results

### 3.1. Baseline Characteristics

A total of 12 GP practices participated, with 1 not submitting data at t1. The age of the participating GPs ranged from 36 to 62 years. Complete data were available for 68 patients (Table 1) and included in the following analysis. Patients were, on average, 78.1 (SD = 6.9, 65–94) years old, and 38.2% were women. No major differences in education level between the IG and CG were observed. The groups differed in terms of the number of disease categories mentioned. The IG had 15 (44%) patients with 8–10 diseases in 14 categories, whereas the CG consisted of only 4 (12%) patients. At t0, the median number of GP-reported drugs was 8.0 per medication list in the CG and 7.0 in the IG. The median number of patient-reported drugs was 7.5 in the CG and 7.0 in the IG (Table 1).

### 3.2. Cross-Sectional Analysis at t0

Cross-sectional analysis at t0 showed similar results for the IG and CG for drug name agreement (Table 2). The median percentage agreement at t0 regarding the number of documented drugs was 72.1% for the IG and 73.9% for the CG. The difference between the groups was not statistically significant (Appendix A). When the reported drug strength was included, the agreement was 66.7% for the IG and 60.0% for the CG. Table 2 shows the results of the sensitivity analysis excluding OTC drugs. The agreement on drug name was 80.0% for the IG and 76.4% for the CG. The maximum difference between the agreement, including and excluding OTC drugs, was found in the IG (Table 2). When analyzing the gender subgroups, differences were observed between men and women (Table 2). At baseline, women had over 15% less relative agreement on reported drugs than men.

### 3.3. Longitudinal Analysis

**Figure 1:**
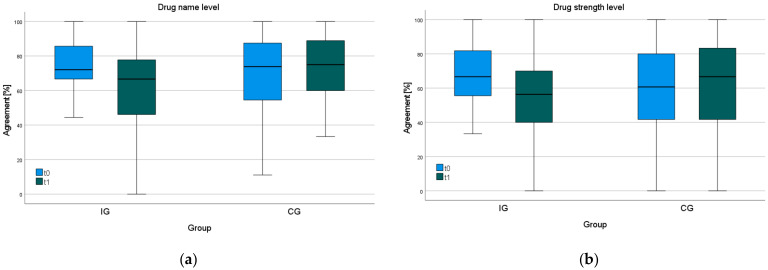
(**a**) Percentage agreement of IG and CG based on reported drug name. (**b**) Percentage agreement of IG and CG based on reported drug name and strength.

**Figure 2:**
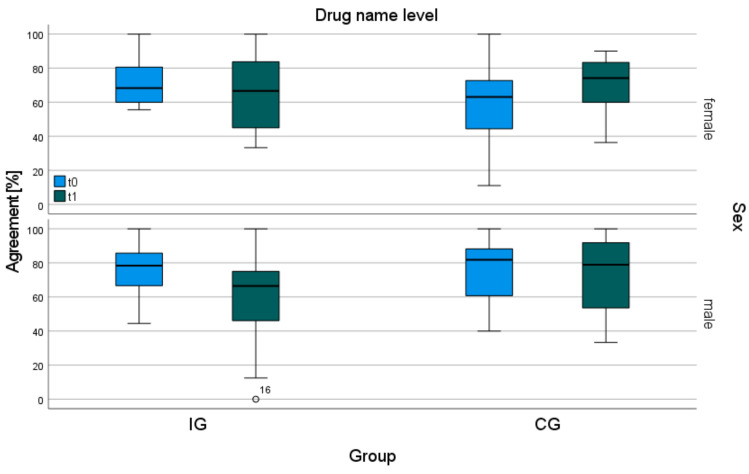
Percentage agreement of gender subgroups of IG and CG based on reported drug name.

There were no statistically significant changes in the number of GP- or patient-reported drugs after six months of follow-up (Table 1). The agreement analysis of drug names showed no statistically significant difference between the groups after six months (Appendix A). The median agreement of the IG decreased statistically significantly from 72.1% to 66.7% (*p* = 0.017), while the median agreement of the CG remained unchanged (t0 = 73.9%, t1 = 75.0% (*p* > 0.05)) (Figure 1, Table 2). The decrease in agreement related to the reported drug name and strength within the IG showed a similar tendency. In 3.9% of cases, drug strength was missing. The sensitivity analysis excluding OTC drugs showed over the time of the intervention, in a similar way as before, a decrease in agreement on reported drug names in the IG (from 80.0% to 69.0% *p* = 0.023). The control group’s agreement remained unchanged at 76.4% (Figure 1, Table 2). After six months of intervention, the median agreement of men in the IG decreased statistically significantly from 78.4% to 66.4% (*p* = 0.014) (Figure 2, Table 2). The agreement of women in the CG was 63.1% at t0 and 74.2% (*p* > 0.05) at t1, and in the IG, it was 68.3% at t0 and 66.7% (*p* > 0.05) at t1 (Figure 2, Table 2).

### 3.4. Differences in ATC Groups

Drugs with 100% agreement at baseline were primarily from the cardiovascular drug group (ATC group: C), including bisoprolol, valsartan, spironolactone, sacubitril, and rivaroxaban (Appendix B). Metformin from ATC group A (alimentary tract and metabolism) and levothyroxine from group H (systemic hormonal preparations) also had 100% agreement. Metamizole as a prescription analgesic had an agreement of only 29.4%. In contrast, a non-prescription drug such as acetylsalicylic acid, used as an analgesic with 500 mg strength and as an antiplatelet agent with 100 mg strength, had an agreement of 94.7%. For on-demand medication, such as formoterol used in case of an asthma attack, an agreement of 54.6% was found.

At baseline, ATC groups B (blood and blood-forming organs), C (cardiovascular system), and H (hormone preparation system) exhibited the highest level of agreement (B: 90.5%, C: 87.1%, H: 91.6%), reflecting the analysis of bisoprolol, valsartan, spironolactone, and sacubitril in group C, rivaroxaban and apixaban in group B, and levothyroxine in group H (Table 3). Group N (nervous system), on the other hand, exhibited the lowest level of agreement (N: 42.9%). The most frequently prescribed drugs in this group were prescription analgesics and antidepressants, with 27.1% of patients taking drugs that were not included in the GP’s medication plan. The proportion of deletions, i.e., medication reported by the GP but not by the patient, was 30.0%. Group Z (over-the-counter drugs) had the highest proportion of additions that the GP was not informed about, at 38.4%. Group C had a high level of disagreement on absolute terms (12 (5.0%) additions and 19 (7.9%) deletions), but this was lower for the total number of reported drugs. The most frequent ATC groups that the patient did not report were group S (sensory organs) at 42.9%, R at 30%, and N also at 30%. The distribution of additions, deletions, and agreements of drugs in ATC groups at t1 was similar to that at baseline (Table 3).

## 4. Discussion

This exploratory analysis of agreement was intended to test the feasibility of this evaluation and provide initial insights into what results can be expected. The results of this analysis illustrate the extent of inconsistencies in the reported drug data. In both study groups, the median agreement ranged from 72% to 80%, independent of the time point, including or excluding OTC drugs. In nearly all cases, the median agreement including drug strength was 10% lower compared to the median agreement on the reported drug name. No statistically significant improvement in agreement was found after the intervention using a paper-based patient portfolio. At t0, the IG had a higher prevalence of comorbidities compared to the CG. However, the groups did not differ in the number of reported drugs or agreement at t0. Differences in gender subgroups were found in both groups. The agreement between male patients and GPs worsened after the study period, whereas women’s agreement seemed to improve in the CG. Furthermore, it was discovered that ATC groups N, R, and Z had the lowest agreement rate. Metamizole showed the lowest agreement of the drugs analyzed, whereas bisoprolol and valsartan from ATC group B showed 100% agreement.

The findings of disagreement between patients and GPs about the reported drug data align with study expectations. Due to the large number of drugs (>5) taken by patients, a high risk of missing or incorrect information was expected. Our results match the findings of a previous study, where discrepancies between data reported by the GP, patient, and electronic medication files on the omission of drug, dose, drug strength, and formulary were found [27]. These differences in the reported medication could cause medical errors or undetected drug–drug interactions for the patient [28].

An unexpected statistically significant decrease in agreement in the IG, which was also present in the sensitivity analysis when excluding OTC drugs, was observed. The participatively developed portfolio required a high degree of patient motivation due to the amount of information that patients must record. Furthermore, due to time and staff shortages, one practice in the IG did not report all drugs at t1. However, the patient portfolio must be adapted for a further main study. To improve feasibility, the portfolio must be more concise.

At t0, the percentage agreement of men in the IG and CG was higher than that of women. This lower agreement rate in women could be due to a higher treatment burden (TB) in women, which has also been observed for women with atrial fibrillation [29]. Treatment burden is defined as the “workload of healthcare” and includes issues such as understanding health information, scheduling medication, and visiting the doctor [30]. Other international studies have also shown a higher incidence of TB in women than in men with chronic illnesses [31,32]. Interestingly, women’s agreement increased over the time of the intervention in the CG, while men showed a decreasing tendency in agreement. This may reflect women’s better health behavior and greater interest in health information compared to men [33]. Due to the uneven gender distribution between the groups, the observed differences between genders could, on the other hand, be coincidental.

Differences in agreement between drug groups were found. ATC groups B (blood and blood-forming organs) and C (cardiovascular system) demonstrated a high level of agreement, particularly on the total number of reported drugs. These drugs are typically part of long-term medication, indicating that patients adhere closely to their medication plan, are sensitive to these drugs, and inform their GP of any changes. This result is also reflected in the agreement of individual drugs from groups B and C. Consistent with our findings, similar results have shown a high level of agreement for cardiovascular drugs [9]. Another study also found a high level of agreement between patient reports and computerized pharmacy records for antihypertensive drugs [34]. In contrast, groups N (nervous system), R (respiratory tract), and Z (over-the-counter drugs) showed only approximately 50% agreement. Group N primarily consisted of Rx analgesics, indicating that in the past seven days, patients often consume drugs that are not listed on their GP’s medication plan, that they forget to report these analgesics, or that they do not take the analgesics listed in the GP’s medication plan. Additionally, seeing different specialists, such as orthopedists, rheumatologists, or neurologists, can contribute to this disagreement. In this context, it is interesting to note that metamizole, as an API of group N, showed a high rate of additions, although preparations with this active ingredient are only available on prescription in Germany. Again, this aligns with previous findings, where drugs from the central nervous system group also showed less than 40% agreement [9]. As expected, OTC drugs showed a high rate of additionally reported drugs by the patient. This supports the thesis that patients are not informing their GP about all consumed drugs, particularly when it comes to OTC drugs. This lack of information could lead to drug–drug and drug–disease interactions, as well as medical errors, due to the GP’s resultant inadequate decision-making basis [35,36,37,38].

### Strengths and Limitations

To the best of our knowledge, this is the first analysis of the agreement of reported drugs within a prospective, cluster randomized, multicenter pilot study using a paper-based patient portfolio. Drug data are heterogeneous data with a large amount of different information. Analysis using a self-developed R script allows for a precise comparison of these data. Filtering by specific drug groups and individual drugs is another advantage of this R script. In summary, with this analysis, a method for assessing agreement as an outcome variable for comparing medication data, the intervention, and the study design was developed and tested.

Our results were limited by the complex nature of the patient questionnaire, which included, among many other queries, several questions about specific drug information, such as strength, package size, dose, and year of first use, as well as other health-related questions. Soliciting such varied and extensive information can result in a greater susceptibility to errors due to inaccurate information. On the other hand, if the patient and GP report a different drug strength, this will not necessarily result in an incorrect daily dose. Moreover, GPs sent heterogeneous information about medication data, with some using standardized medication lists (German BMP, or *Bundeseinheitlicher Medikationsplan*) and providing detailed drug information. In some cases, the HCA completed the questionnaire instead of the GP; because all information was taken from the practice management system, we do not believe that this led to inaccuracies. In contrast, others sent incomplete medication plans or used other formats. Consequently, some information, such as the drug strength and, very often, drug dosage, was missing. This was not well compensated for by the small sample size. In future studies, to enable a database as homogenous as possible, a small set of precise questions about drug data should be developed. Data collection was paper-based for patients and electronic for GPs. This may have influenced the transmitted data. Nevertheless, the formulation of questions about drugs was very similar on the patient and GP questionnaires. The advanced age of the patients is another limiting factor which may have influenced the completeness and accuracy of drug information. In light of these circumstances, patients were permitted to complete the questionnaire with the assistance of a family member or caregiver. The GP setting also presented some difficulties. Low staff levels and time capacity may have led to missing data, mainly due to the high volume of information requested. In addition, implementing the intervention may have been difficult for some practices because of the COVID-19 pandemic and the associated challenges.

## 5. Conclusions

This explorative analysis of the abovementioned cRCT pilot study indicates the expected substantial gap between patient- and GP-reported drug data. The paper-based patient portfolio did not have any impact on closing this gap. It is evident that improvements are required in the synchronization of drug data within the primary care setting. Such improvements are necessary to establish a robust information base for decision-making by the GP. The results also reveal how differences in gender and in ATC groups play an important role in agreement. In conclusion, the results of this pilot study indicate that the following would be essential for further evaluations: an improvement in the patient portfolio (e.g., electronic-based), with stratification by gender and ATC groups.

## Figures and Tables

**Table 1:** Baseline practice and patient characteristics.

	CG	IG	*p*
GP practice (n ^3^)	6	5	
Age of GPs (median, IQR ^2^)	55.5 (45.0–60.5)	48.0 (42.5–59.0)	*
Female GP (n ^3^,%)	0 (0)	4 (80)	*
Proportion of group practice (n ^3^,%)	2 (33)	4 (80)	*
Region of Hesse (n ^3^,%)	4 (67)	3 (60)	*
Region of Saxony (n ^3^,%)	2 (33)	2 (40)	*
Patients (n ^3^)	34	34	
Age of patients (mean, SD ^1^)	77.4 ± 6.4	78.8 ± 7.3	0.484
Female patients (n ^3^, %)	10 (29)	16 (47)	0.134
Education level			
Low (n ^3^, %)	13 (38)	7 (21)	0.110
Medium (n ^3^, %)	11 (32)	15 (44)	0.318
High (n ^3^, %)	10 (29)	12 (35)	0.604
Morbidity			
2–4 disease categories (n ^3^, %)	11 (32)	5 (15)	0.086
5–7 disease categories (n ^3^, %)	17 (50)	13 (38)	0.329
8–10 disease categories (n ^3^, %)	4 (12)	15 (44)	0.003
11–13 disease categories (n ^3^, %)	2 (6)	1 (3)	0.555
Living arrangement			
Living alone (n ^3^, %)	5 (15)	8 (24)	0.355
Living with other persons (partner/family) (n ^3^, %)	28 (82)	18 (53)	0.010
Assisted living/nursing home (n ^3^, %)	0 (0)	5 (15)	0.020
Unknown (n ^3^, %)	1 (3)	3 (9)	0.303
Number of drugs reported by GP at t0 (median, IQR ^2^)	8.00 (5.75–10.00)	7.00 (5.00–9.25)	0.753
Number of drugs reported by GP at t1 (median, IQR ^2^)	7.00 (6.00–10.00)	7.00 (3.75–9.00)	0.176
Number of drugs reported by patient at t0 (median, IQR ^2^)	7.50 (5.75–10.00)	7.00 (5.00–9.00)	0.574
Number of drugs reported by patient at t1 (median, IQR ^2^)	7.50 (6.00–10.25)	7.50 (5.00–8.25)	0.177

^1^ SD, standard deviation; ^2^ IQR, interquartile range; ^3^ n, number, * no statistics due to small numbers.

**Table 2:** Median percentage agreement of CG and IG based on reported drug name or drug name and strength at baseline t0 and after six-month follow-up t1.

	t0 [%]	t1 [%]	*p*
Agreement of drug name			
CG (median, IQR ^2^)	73.9 (54.2–87.5)	75.0 (59.3–89.2)	0.554
IG (median, IQR ^2^)	72.1 (65.0–85.7)	66.7 (44.6–78.3)	**0.017**
Agreement of drug name and strength			
CG (median, IQR ^2^)	60.0 (41.3–80.8)	66.7 (41.3–83.9)	0.178
IG (median, IQR ^2^)	66.7 (55.6–82.0)	56.3 (40.7–71.3)	**0.005**
Agreement of drug name (excluding OTC ^1^)			
CG (median, IQR ^2^)	76.4 (56.3–85.7)	76.4 (61.2–90.2)	0.207
IG (median, IQR ^2^)	80.0 (66.7–93.8)	69.0 (50.0–80.5)	**0.023**
Agreement of drug name and strength (excluding OTC ^1^)			
CG (median, IQR ^2^)	61.3 (41.3–80.8)	68.3 (40.0–82.8)	0.094
IG (median, IQR ^2^)	66.7 (54.2–83.9)	60.0 (45.2–75.7)	**0.010**
Agreement of drug name in subgroups			
CG women (median, IQR ^2^)	63.1 (41.7–73.3)	74.2 (60.0–83.9)	0.139
CG men (median, IQR ^2^)	81.8 (60.4–88.5)	78.9 (51.8–92.4)	0.781
IG women (median, IQR ^2^)	68.3 (60.0–81.9)	66.7 (42.5–85.6)	0.328
IG men (median, IQR ^2^)	78.4 (66.7–89.3)	66.4 (44.6–75.7)	**0.014**

Agreement was calculated as the absolute agreement divided by the sum of agreements and disagreements. Differences between t0 and t1 were calculated using the Wilcoxon test with α = 5% and CI = 95%. ^1^ OTC, over-the-counter; ^2^ IQR, interquartile range.

**Table 3:** Total numbers of additions, deletions, and agreement in ATC groups.

ATC Group	Additions * t0	Deletions ** t0	Agreement *** t0	Additions * t1	Deletions ** t1	Agreement *** t1
A: alimentary tract and metabolism	13 (16.5%)	16 (20.2%)	50 (63.3%)	15 (18.1%)	17 (20.5%)	51 (61.4%)
B: blood and blood-forming organs	0 (0%)	4 (9.5%)	38 (90.5%)	3 (8.6%)	1 (2.9%)	31 (88.5%)
C: cardiovascular system	12 (5.0%)	19 (7.9%)	208 (87.1%)	24 (10.2%)	20 (8.5%)	192 (81.3%)
D: dermatologicals	1 (100%)	0 (0%)	0 (0%)	0 (0%)	0 (0%)	0 (0%)
G: genitourinary system and sex hormones	2 (10.0%)	2 (10.0%)	16 (80.0%)	5 (25.0%)	3 (15.0%)	12 (60.0%)
H: systemic hormonal preparations	1 (4.2%)	1 (4.2%)	22 (91.6%)	3 (12.0%)	4 (16.0%)	18 (72.0%)
J: systemic anti-infectives	0 (0%)	0 (0%)	1 (100%)	0 (0%)	1 (50.0%)	1 (50.0%)
L: antineoplastic and immunomodulating agents	0 (0%)	0 (0%)	2 (100%)	0 (0%)	1 (33.3%)	2 (66.7%)
M: musculoskeletal system	1 (6.3%)	3 (18.7%)	12 (75.0%)	0 (0%)	2 (11.8%)	15 (88.2%)
N: nervous system	19 (27.1%)	21 (30.0%)	30 (42.9%)	16 (28.6%)	13 (23.2%)	28 (48.2%)
P: antiparasitic products	1 (100%)	0 (0%)	0 (0%)	1 (100%)	0 (0%)	0 (0%)
R: respiratory system	6 (20.0%)	9 (30%)	15 (50.0%)	4 (15.4%)	9 (34.6%)	13 (50.0%)
S: sensory organs	1 (14.2%)	3 (42.9%)	3 (42.9%)	2 (25.0%)	1 (12.5%)	5 (62.5%)
V: variable	0 (0%)	0 (0%)	0 (0%)	0 (0%)	0 (0%)	0 (0%)
Z: over-the-counter drugs	28 (38.4%)	8 (11.0%)	37 (50.6%)	26 (33.3%)	13 (16.7%)	39 (50.0%)

* Additions: Patients reported drugs that are not on the medication list of the GP. ** Deletions: GP-reported drugs that the patient does not report. *** Agreement: Drugs that are reported by the patient as well as the GP. The sum of additions, deletions, and agreements is 100%.

## Data Availability

The data presented in this study are only available on request from the corresponding author due to ethical and legal reasons.

## Abbreviation

API	active pharmaceutical ingredient
ATC	anatomical therapeutic chemical
BMP	Federal standardized medication plan
CG	control group
GP	general practitioner
IG	intervention group
IQR	interquartile range
OTC	over-the-counter
PZN	German pharmaceutical registration number
Rx	prescription-only
SD	standard deviation
TB	treatment burden
t0	baseline
t1	follow-up after 6 months

## Appendix A

**Table A1:** The median percentage agreement of the CG and IG due to the reported drug name or drug name and strength, at baseline t0 and after the six-month follow-up t1, and the corresponding *p*-value.

	CG [%] (Median, IQR ^2^)	IG [%] (Median, IQR ^2^)	*p*
Agreement of drug name			
Baseline (t0)	73.9 (54.2–87.5)	72.1 (65.0–85.7)	0.667
Follow-up (t1)	75.0 (59.3–89.2)	66.7 (44.6–78.3)	0.079
Agreement of drug name and strength			
Baseline (t0)	60.0 (41.3–80.8)	66.7 (55.6–82.0)	0.243
Follow-up (t1)	66.7 (41.3–83.9)	56.3 (40.7–71.3)	0.199
Agreement of drug name (excluding OTC ^1^)			
Baseline (t0)	76.4 (56.3–85.7)	80.0 (66.7–93.8)	0.306
Follow-up (t1)	76.4 (61.2–90.2)	69.0 (50.0–80.5)	0.196
Agreement of drug name and strength (excluding OTC ^1^)			
Baseline (t0)	61.3 (41.3–80.8)	66.7 (54.2–83.9)	0.134
Follow-up (t1)	68.3 (40.0–82.8)	60.0 (45.2–75.7)	0.302

Agreement was calculated as the absolute agreement divided by the sum of agreements and disagreements. Group differences were calculated using the Mann–Whitney U test with α = 5% and CI = 95%. ^1^ OTC, over-the-counter; ^2^ IQR, interquartile range.

## Appendix B

**Table A2:** The agreement of single APIs and the corresponding ATC group based on the PZN or drug strength.

API	ATC Group	Agreement [%]
Bisoprolol	C	100.00
Levothyroxine	H	100.00
Metformin	A	100.00
Valsartan	C	100.00
Spironolactone	C	100.00
Rosuvastatin	C	100.00
Sacubitril	C	100.00
Rivaroxaban	C	100.00
Acetylsalicylic acid	B	94.74
Apixaban	B	90.00
Atorvastatin	C	89.47
Ramipril	C	89.47
Torasemide	C	89.29
Metoprolol	C	82.35
Candesartan	C	81.82
Hydrochlorothiazide	C	81.82
Amlodipine	C	80.95
Simvastatin	C	78.57
Tamsulosin	G	75.00
Edoxaban	B	71.43
Allopurinol	M	66.67
Beclometasone	R	66.67
Ezetimibe	C	66.67
Colecalciferol	A	65.22
Pantoprazole	A	60.00
Insulin	A	60.00
Formoterol	R	54.55
Metamizole	N	29.41

The percentage agreement of the API is calculated by matching patient- and GP-reported drug APIs in relation to the sum of agreements and disagreements.
